# Contribution of BubR1 to oxidative stress-induced aneuploidy in p53-deficient cells

**DOI:** 10.1002/cam4.101

**Published:** 2013-06-26

**Authors:** Ayae Ikawa-Yoshida, Koji Ando, Eiji Oki, Hiroshi Saeki, Ryuichi Kumashiro, Kenji Taketani, Satoshi Ida, Eriko Tokunaga, Hiroyuki Kitao, Masaru Morita, Yoshihiko Maehara

**Affiliations:** 1 Departments of Surgery and Science Graduate School of Medical Sciences, Kyushu UniversityFukuoka, Japan; 2 Molecular Oncology Graduate School of Medical Sciences, Kyushu UniversityFukuoka, Japan; 3 Center for Integration of Advanced Medicine, Life Science and Innovative Technology, Kyushu UniversityFukuoka, Japan; 4 Molecular Targeting Therapy Graduate School of Medical Sciences, Kyushu UniversityFukuoka, Japan

**Keywords:** Aneuploidy, BubR1, gastric cancer, oxidative stress, p53

## Abstract

DNA aneuploidy is observed in various human tumors and is associated with the abnormal expression of spindle assembly checkpoint (SAC) proteins. Oxidative stress (OS) causes DNA damage and chromosome instability that may lead to carcinogenesis. OS is also suggested to contribute to an increase in aneuploid cells. However, it is not clear how OS is involved in the regulation of SAC and contributes to carcinogenesis associated with aneuploidy. Here we show that an oxidant (KBrO_3_) activated the p53 signaling pathway and suppressed the expression of SAC factors, BubR1, and Mad2, in human diploid fibroblast MRC5 cells. This suppression was dependent on functional p53 and reactive oxygen species. In p53 knockdown cells, KBrO_3_ did not suppress BubR1 and Mad2 expression and increased both binucleated cells and cells with >4N DNA content. BubR1 and not Mad2 downregulation suppressed KBrO_3_-induced binucleated cells and cells with >4N DNA content in p53 knockdown cells, suggesting that BubR1 contributes to enhanced polyploidization by a mechanism other than its SAC function. In analysis of 182 gastric cancer specimens, we found that BubR1 expression was significantly high when p53 was positively stained, which indicates loss of p53 function (*P *=* *0.0019). Moreover, positive staining of p53 and high expression of BubR1 in tumors were significantly correlated with DNA aneuploidy (*P *=* *0.0065). These observations suggest that p53 deficiency may lead to the failure of BubR1 downregulation by OS and that p53 deficiency and BubR1 accumulation could contribute to gastric carcinogenesis associated with aneuploidy.

We found that OS could contribute to the emergence of polyploid cells when p53 was deficient in normal human fibroblast cells. Importantly, this polyploidization could be suppressed by downregulating the expression of one spindle assembly checkpoint factor, BubR1. We also found that p53 dysfunction and BubR1 accumulation strongly correlate with the extent of aneuploidy in gastric cancer specimen and our data suggest that p53 deficiency and BubR1 accumulation could contribute to gastric carcinogenesis associated with aneuploidy.

## Introduction

In 1891, von Hansemann et al. 1 first observed a numerical chromosome aberration, DNA aneuploidy, in malignant tumors. It has been previously revealed that significant portions of tumors exhibit DNA aneuploidy and tumors with DNA aneuploidy are significantly correlated with poor cancer prognosis 2. Although extensive efforts have been made to unveil the mechanism underlying DNA aneuploidy, the mechanism is still not completely understood.

The appropriate expression of spindle assembly checkpoint (SAC) factors may be an important factor for maintaining chromosome stability. SAC is a surveillance system controlling the segregation of sister chromatids to daughter cells in mitosis 3. BubR1 and Mad2 are SAC factors that regulate CDC20 to activate the E3 ubiquitin ligase called anaphase-promoting complex/cyclosome (APC/C). APC/C targets securin and cyclin B for degradation and is required for metaphase–anaphase transition 4. When kinetochores are not fully occupied by microtubules or when the tension between sister chromatids is uneven, SAC inhibits CDC20; this delays the metaphase–anaphase transition until all kinetochores are attached to microtubules in an appropriate manner 5. Overexpression or downregulation of SAC factors may cause aberrant SAC functioning, an unequal segregation of chromosomes, and DNA aneuploidy 6–10. DNA aneuploidy is often observed in various tumors with abnormal expression of SAC factors 11.

The p53 signaling pathway is a major suppressor of chromosome instability 12. p53 controls the transcription of cell cycle checkpoint factors such as p21 and regulates cell cycle progression at G1–S or G2–M transitions 13. In addition, p53 regulates BubR1 and Mad2 expression and suppresses centrosome amplification 14–15 or aneuploidy. The correlation between an abnormal p53 status and DNA aneuploidy has been observed in various human tumors 9–16.

Aerobic metabolism, with its advantage of high levels of energy production, is essential for all organisms exposed to oxidative stress (OS). However, OS causes DNA damage and is an indirect cause of mutations, gene deletions, and chromosome instability that may lead to malignant transformation. OS is also suggested to contribute to an increase in aneuploid cells 17–18. However, the mechanism by which the p53 signaling pathway activated by OS suppresses aneuploidy is not completely understood. In this study, we found that BubR1 and Mad2 are downregulated by OS in a p53-dependent manner. When p53 expression was suppressed by small interfering RNA (siRNA), BubR1 and Mad2 downregulation by OS was not observed and polyploid cells increased. Importantly, BubR1 and not Mad2 downregulation suppressed OS-induced polyploidization in p53 knockdown cells, suggesting that BubR1 could have novel functions other than SAC that contribute to polyploidization. Moreover, analysis of clinical gastric cancer specimens revealed that tumors with positive staining for p53 and high expression of BubR1 tended to exhibit aneuploidy. Our findings could provide one possible model for the mechanism underlying gastric carcinogenesis associated with DNA aneuploidy.

## Material and Methods

### Reagents and antibodies

Potassium bromate (KBrO_3_) was obtained from Sigma-Aldrich (Gillingham, U.K.). The antibodies used were as follows: rabbit polyclonal anti-phospho(ser15)-p53 (Cell Signaling Technology, Beverly, MA), mouse monoclonal anti-p53 (Dako Cytomation, Glostrup, Denmark), mouse monoclonal anti-p21 (Dako Cytomation), mouse monoclonal anti-BubR1 (Clone 9; BD Transduction Laboratories, San Jose, CA), mouse monoclonal anti-Mad2 (ImmuQuest, Cleveland, U.K.), mouse monoclonal anti-β-actin (Sigma-Aldrich, St. Louis, MO), mouse monoclonal anti-lamin A/C (Cell Signaling Technology), rat monoclonal anti-α-tubulin (Abcam, Cambridge, U.K.), Alexa 594-conjugated goat anti-rat IgG (Invitrogen, Carlsbad, CA), and Alexa 488-conjugated goat anti-mouse IgG (Invitrogen).

### Cell culture

Human fibroblast cells (MRC5) and the gastric cancer cells SNU-1 and KATOIII were obtained from ATCC (Manassas, VA). The gastric cancer cells MKN-45 and MKN-28 were obtained from the Riken Cell Bank (Tsukuba, Japan). MRC5 cells were cultured in Eagle's minimum essential medium supplemented with fetal bovine serum (10% v/v), at 37°C in a 5% CO_2_ environment. We used MRC5 cells collected between passages 5 and 8. SNU-1, KATOIII, MKN-45, and MKN-28 cells were cultured in Roswell Park Memorial Institute 1640 medium supplemented with fetal bovine serum (10% v/v) at 37°C in a 5% CO_2_ environment.

### Western blotting

Cells in the logarithmic growth phase were lysed in lysis buffer (0.5% NP-40, 20 mmol/L Tris-HCl [pH 8.0], 150 mmol/L NaCl, 1 mmol/L EDTA, and 1% protease inhibitor cocktail [Nacalai Tesque, Inc., Kyoto, Japan]). The protein content was determined using the Bradford assay (Bio-Rad, Hercules, CA). The same amount of protein (8 μg) was loaded in each lane. The immunoreactive bands were detected using the LAS3000 System (GE Healthcare, Tokyo, Japan).

### Quantification of mRNA in fibroblast cells using real-time PCR

Total RNA was isolated using ISOGEN (Nippongene, Tokyo, Japan), and complementary DNAs (cDNAs) were synthesized from RNAs using SuperScript™ III First-Strand Synthesis SuperMix (Invitrogen) according to the manufacturer's instructions. Quantitative polymerase chain reaction (PCR) amplification was performed using the Applied Biosystems StepOnePlus™ Real-Time PCR System (Life Technologies, Tokyo, Japan) and the QuantiFast^™^ SYBR^®^ Green PCR Kit (QIAGEN, Hilden, Germany). BubR1 and Mad2 transcript levels were determined using β-actin as an endogenous control. The oligodeoxynucleotide primers were as follows: BubR1, 5′-CTCGTGGCAATACAGCTTCA-3′ (forward) and 5′-CTGGTCAATAGCTCGGCTTC-3′ (reverse) 9; Mad2, 5′- ACTTAAATATCTCCCTACCTATACTGAGTCAA-3′ (forward) and 5′-TAGTAACTGTAGATGGAAAAACTTGTGCTA-3′ (reverse) 19; and β-actin, 5′-CCACGAAACTACCTTCAAC-3′ (forward) and 5′-GATCTTCATTGTGCTGGG-3′ (reverse) 9.

### siRNA transfection

MRC5 cells were transfected with 10 nmol/L siRNA oligonucleotides using Lipofectamine RNAiMAX Reagent (Invitrogen) according to the manufacturer's instructions. The siRNAs used were as follows: p53 (5′-GAAAUUUGCGUGUGGAGUA-3′, ON-TARGETplus human TP53 siRNA; Dharmacon, Lafayette, CO), BubR1 (5′-AAGGGAAGCCGAGCUGUUGAC-3′) 20, and Mad2 (SMARTpool ON-TARGETplus MAD2L1 siRNA; Dharmacon). The ON-TARGETplus Non-Targeting Pool (Dharmacon) was used as a control. Cells were lysed for Western blotting 24–36 h after transfection.

### Flow cytometry

Cells were trypsinized, washed with cold phosphate-buffered saline (PBS), and fixed with 70% ethanol in PBS at −20°C. Samples were washed with cold PBS, resuspended in propidium iodide solution (50 μg/mL) containing RNase A (1 mg/mL), and incubated at 37°C for 30 min. The nuclear DNA content of 10^4^ cells was analyzed using the FACS Calibur System (Becton Dickinson) with CellQuest software (BD Biosciences, Japan).

### Fluorescence immunostaining

Cells were fixed with 4% paraformaldehyde and immunostained with antibodies against lamin A/C and α-tubulin. Double staining was performed with Alexa 488- and Alexa 594-conjugated secondary antibodies, and the nuclei were counterstained with 4′,6-diamino-2-phenylindole (DAPI). Images were captured using a BIOREVO BZ-9000 fluorescence microscope (Keyence, Tokyo, Japan). We determined cells as binucleate on the basis of the structure of the microtubules and nuclear matrix.

### Patients

A total of 182 randomly selected Japanese patients with primary gastric cancer, who underwent a gastrectomy between 1994 and 2006 at the Department of Surgery and Science, Graduate School of Medical Sciences, Kyushu University Hospital, Fukuoka, Japan, were included in this study. None of the patients had been preoperatively treated with cytotoxic drugs. Informed consent was obtained from each patient.

### Immunohistochemical staining

Immunohistochemical staining was performed according to a previously described protocol 9. In brief, the sections were placed in 10 mmol/L citrate buffer (pH 6.0) and boiled in a microwave for epitope retrieval. Endogenous peroxidase activity was quenched by incubation in 0.3% H_2_O_2_. After blocking with 10% goat serum in PBS, the sections were incubated with a primary antibody. Streptavidin–biotin–peroxidase staining was performed using the Histofine SAB-PO (M) Immunohistochemical Staining Kit (Nichirei, Tokyo, Japan), according to the manufacturer's instructions. Sections were counterstained with Mayer's hematoxylin and examined at a magnification of 400×.

The p53 expression status was classified by examining immunostaining intensity as described previously 21–22. A distinct nuclear immunoreactivity for p53 was recorded as positive, and the nuclear staining pattern was usually diffuse. For tumors that showed heterogeneous staining, the predominant pattern was used for scoring. Specimens with less than 10% positively stained cancer cell nuclei were defined as negative, and the remainder were defined as positive (Fig. S1).

The BubR1 expression status was analyzed by examining immunostaining intensity as described previously 9. The BubR1 expression level in lymph follicles, which were equally stained in all samples, was assigned an expression score of 1. Weaker staining was assigned a score of 0, and samples with stronger staining than that in the follicles were assigned a score of 2 (Fig. S1).

### Analysis of DNA ploidy by laser scanning cytometry

Nuclear DNA content of gastric cancer samples was measured using a laser scanning cytometer (CompuCyte, Westwood, MA) as described previously 23. The samples were obtained from the same paraffin-embedded blocks as those used for immunohistochemical staining. A DNA content histogram was generated, and DNA ploidy was determined. The DNA index (DI) was calculated according to previously published principles 24. The nuclei were examined after each scan to exclude debris and attached nuclei from analysis. The DI of G0/G1-phase lymphocytes or fibroblasts was used as the reference DI = 1.0. Tumors with a DI < 1.2 were defined as diploid, whereas DI ≥ 1.2 or multi-indexed samples were defined as aneuploid.

### Statistical analysis

Statistical analysis was performed using the JMP 8.0 statistical software package (SAS Institute, Cary, NC). The Student's *t*-test and Pearson's chi-square test were used where appropriate.

## Results

### OS activated the p53 signaling pathway and suppressed BubR1 and Mad2 expression

**Figure 1:**
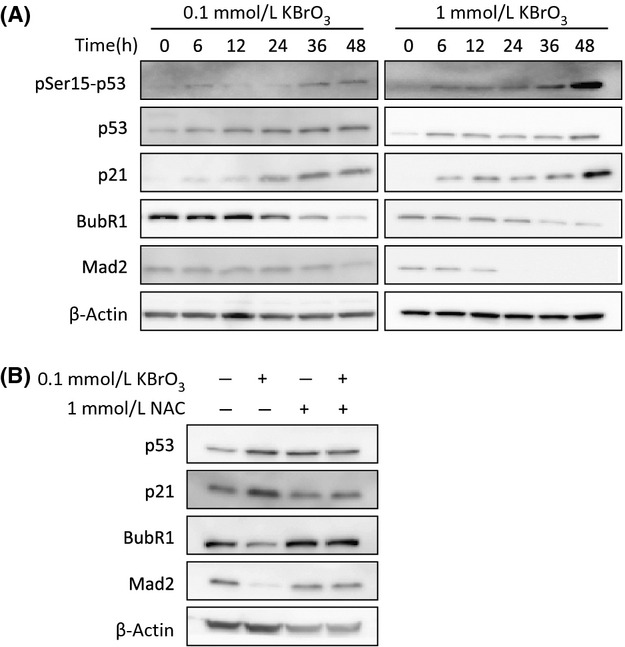
OS induced the p53 signaling pathway and suppressed the expression of BubR1 and Mad2. (A) MRC5 cells were exposed to 0.1 or 1 mmol/L KBrO_3_ for 0 to 48 h. Each protein was analyzed by Western blotting. (B) MRC5 cells were incubated with 1 mmol/L of the ROS scavenger *N*-acetylcysteine (NAC) 15 min prior to treatment with 0.1 mmol/L KBrO_3_. After 48 h of incubation, cells were collected and analyzed by Western blotting. β-actin served as a loading control.

Previous studies indicated that p53 could regulate BubR1 expression 14–15. To examine the relationship between the OS-activated p53 signaling pathway and BubR1 expression, we cultured human diploid fibroblast MRC5 cells in the presence of the oxidant KBrO_3_ for as long as 48 h and analyzed the p53 signaling pathway and BubR1 expression by Western blotting. As reported previously 25, both a low concentration (0.1 mmol/L) and a high concentration (1 mmol/L) of KBrO_3_ induced the phosphorylation of p53 Ser15 and accumulation of p53 and p21 proteins (Fig. 1A). Concomitantly, KBrO_3_ suppressed BubR1 expression (Fig. 1A). To examine whether reactive oxygen species (ROS) were responsible for these events, we added the ROS scavenger *N*-acetylcysteine (NAC) before the MRC5 cells were exposed to 0.1 mmol/L KBrO_3_. Neither KBrO_3_-induced accumulation of p53 and p21 proteins nor a decrease in BubR1 protein was observed in the presence of NAC (Fig. 1B). The expression of Mad2, another SAC protein, was also downregulated by KBrO_3_ (Fig. 1A), and Mad2 downregulation was also abolished by NAC (Fig. 1B). These observations indicate that OS activated the p53 signaling pathway and suppressed the expression of BubR1 and Mad2 through the action of ROS.

### OS-induced downregulation of BubR1 and Mad2 expression was p53 dependent

**Figure 2:**
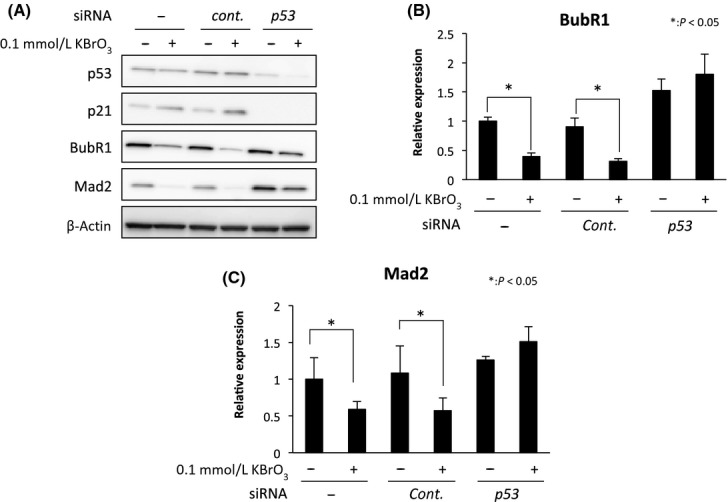
OS-induced downregulation of BubR1 and Mad2 expression was p53 dependent. MRC5 cells were transfected with control siRNA or p53 siRNA, and after 24 h of incubation, 0.1 mmol/L KBrO_3_ was added. (A) Western blot analysis of p53, p21, BubR1, and Mad2 in MRC5 cells 48 h after of KBrO_3_ addition and the relative expression of (B) BubR1 and (C) Mad2 by real-time PCR are shown. p53 knockdown increased the expression of BubR1 and Mad2 mRNAs under OS. The expression scores were normalized to β-actin. Expression levels are relative to those of the nontransfected non-OS cells. Data are shown as means ± SD of triplicate measurements.

To examine the relevance of the p53 signaling pathway for the decrease in BubR1 and Mad2 expression under OS, we used MRC5 cells in which p53 had been knocked down by specific siRNA before exposure to KBrO_3_. p53 knockdown inhibited p21 protein expression (Fig. 2A). Under these conditions, the decrease in BubR1 and Mad2 proteins under OS was not observed (Fig. 2A). OS-induced downregulation of BubR1 and Mad2 was also detected at the messenger RNA (mRNA) level, and p53 knockdown abrogated BubR1 and Mad2 mRNA downregulation (Fig. 2B and C). We also observed downregulation of BubR1 by KBrO_3_ in p53-proficient gastric cancer cell lines (MKN45 and SNU-1) but not in p53-mutant and p53-null gastric cancer cell lines (MKN28 and KATOIII, respectively) (Fig. S2). These results suggest that the decrease in BubR1 and Mad2 expression under OS was dependent on the p53 signaling pathway.

### Suppression of BubR1 expression prevented OS-induced polyploidization in p53-depleted cells

**Figure 3:**
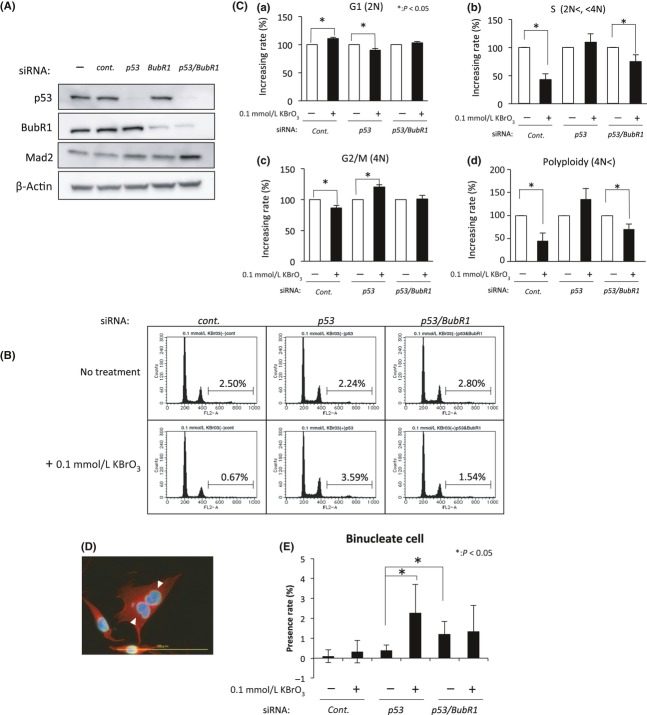
Suppression of BubR1 expression prevented OS-induced polyploidization in p53-depleted cells. (A) MRC5 cells were transfected with control (cont.) siRNA, p53 siRNA, and BubR1 siRNA, and after 36 h of incubation. siRNA-mediated downregulation of p53 and BubR1 was confirmed by Western blotting. (B) To determine the cell cycle distribution, MRC5 cells were transfected with control siRNA, p53 siRNA, and BubR1 siRNA, and after 36 h of incubation, 0.1 mmol/L KBrO_3_ was added. DNA content was analyzed by flow cytometry. The percentages of cells with >4N DNA content are shown. (C) The relative percentages of 0.1 mmol/L KBrO_3_-treated cells in the G1 phase (2N; a), S phase (>2N, <4N; b), G2/M phase (4N; c), and polyploidy (>4N; d) are shown as filled black bars; *P > 0.05. Data are shown as means ± SD of triplicate measurements. (D) A binucleate p53 knockdown MRC5 cell with KBrO_3_ exposure (nuclei indicated by arrowheads) and a merged immunofluorescence image of α-tubulin (red), lamin A/C (green), and DAPI (blue) are shown; scale bar, 100 μm. (E) Percentages of binucleate cells are shown; *P > 0.05.

Dysfunction of the p53 signaling pathway is strongly correlated with aneuploidy 26. We used flow cytometry to analyze the DNA content of MRC5 cells that had been transfected with p53 and/or BubR1 siRNA and exposed to 0.1 mmol/L KBrO_3_ (Fig. 3). siRNA-mediated downregulation of p53 and BubR1 was confirmed by Western blotting (Fig. 3A). In the untreated or control siRNA-treated MRC5 cells, polyploid cells (cells with >4N DNA content) were significantly decreased when the cells were cultured in the presence of 0.1 mmol/L KBrO_3_ (*P *<* *0.05; Fig. 3B and C-d). These results were probably caused by the OS-induced, p53-dependent accumulation of cells in the G1 phase and the decrease in cells in the S to G2/M phases (Fig. 3C-a, -b, and -c). In contrast, when p53 expression was knocked down by siRNA, accumulation in the G1 phase, a decrease in cells in the S to G2/M phases, or a decrease in polyploid cells was not observed under the same conditions (Fig. 3B and C). Polyploid cells are usually generated when DNA synthesis occurs without proper cell division. To identify the events leading to the generation of polyploid cells in the presence of 0.1 mmol/L KBrO_3_ in p53 knockdown cells, we observed the nuclear structure of KBrO_3_-treated cells by fluorescence microscopy. A significant increase in binucleate cells was detected with exposure to KBrO_3_ in p53 knockdown cells (Fig. 3D and E), but not in control siRNA-treated cells (Fig. 3E). When p53 and BubR1 expression was knocked down simultaneously, decreases in S-phase and polyploid cells with KBrO_3_ were observed (Fig. 3C-b and -d). Consistent with this finding, KBrO_3_ did not increase the number of binucleate cells under these conditions (Fig. 3E). Interestingly, in contrast, Mad2 knockdown did not suppress the emergence of polyploid cells with 0.1 mmol/L KBrO_3_ in p53-depleted cells (Fig. S3). These results indicate that BubR1 could be one of the essential factors for OS-induced polyploidization in p53-depleted cells. Our results also suggest that the function of BubR1 required for OS-induced polyploidy may not be related to its SAC function, which is largely mediated by Mad2.

### p53 and BubR1 expression status was related to DNA aneuploidy in gastric cancer specimens

Our data from in vitro experiments indicated that BubR1 is involved in OS-induced polyploidization in p53-deficient cells. Polyploid cells are known to result in aneuploidy and promote tumor development 27,28. We chose gastric cancer to examine whether the correlations among p53 dysfunction, BubR1 expression, and OS-induced aneuploidy were observed in clinical tumor specimens because the gastric mucosa is constantly exposed to strong acid and OS may play an important role in gastric organic disorders, including cancer 30. We immunohistochemically stained p53 and BubR1 in 182 gastric cancer specimens as described previously 9–22 and examined the correlation between them. We regarded samples with p53-positive staining as p53 functional loss samples as described previously 22–33. Among 71 specimens with p53-negative staining, 45 specimens (63.4%) showed high BubR1 expression (score: 1, 2). In contrast, among 111 specimens with p53-positive staining, 95 specimens (85.6%) showed high BubR1 expression (*P *<* *0.01; Table 1). These results suggest that BubR1 tended to be highly expressed when p53 function was lost in gastric cancer.

**Table:** p53 expression status and BubR1 expression level in gastric cancer

BubR1 expression level	p53 staining			*P*-value
	Negative	Positive		
Low	26 (36.6)	16 (14.4)	0.0019*
High	45 (63.4)	95 (85.6)	

The values in parentheses are expressed in %.

* *P* 0.01.

Next, we investigated the relationship between p53/BubR1 expression and DNA aneuploidy in gastric cancer. Using a laser scanning cytometer, we analyzed the DNA content of 77 specimens: 13 specimens with p53-negative staining and low BubR1 expression (score: 0) and 64 specimens with p53-positive staining and high BubR1 expression (score: 1, 2). Among 44 specimens that exhibited aneuploidy, 41 specimens (93.2%) showed p53-positive staining and high BubR1 expression (*P *<* *0.01; Table 2). This significant correlation between p53 dysfunction and high expression of BubR1 and DNA aneuploidy in gastric cancer may support our results from in vitro experiments.

**Table:** DNA ploidy and BubR1/p53 expression status in gastric cancer

p53/BubR1 status	DNA ploidy			*P*-value
	Diploidy	Aneuploidy		
Negative/Low	10 (30.3)	3 (6.8)	0.0065*
Positive/High	23 (69.7)	41 (93.2)	

The values in parentheses are expressed in %.

* *P* 0.01.

## Discussion

It has been proposed that OS may contribute to carcinogenesis by increasing the frequency of genetic mutations and that ROS act as second messengers in multiple intracellular pathways, resulting in malignant transformation 34. Possible models that connect DNA aneuploidy with OS have been proposed 18–35. However, the role of OS in DNA aneuploidy remains controversial. In our in vitro analysis using normal human fibroblast MRC5 cells, when p53 expression was suppressed, OS-producing KBrO_3_ increased the number of polyploid cells, which was partially dependent on sustained BubR1 expression (Fig. 3). Consistent with this finding, we found that the coexistence of p53 dysfunction and high expression of BubR1 strongly correlated with the extent of aneuploidy in gastric cancer specimens (Table 2).

We found that OS activated the p53 signaling pathway and downregulated BubR1 and Mad2 expression (Fig. 1). BubR1 and Mad2 downregulation by OS was p53 dependent (Figs. 2 and S2). During SAC activation, BubR1 and Mad2 form a complex with Bub3 and CDC20 and inhibit the proteasome activity of APC/C 36. The accumulation of BubR1 and Mad2 in the absence of functional p53 may cause the accumulation of APC/C substrates (cyclin A/B, securin, aurora-A, and Plk1) and may evoke some mitotic errors that induce chromosomal aberrations such as polyploidy 7–39. p53 could play a critical role in the suppression of OS-initiated mitotic errors through downregulation of SAC factors. In addition, cells lacking p53 function could have an increased ability to re-enter the cell cycle and initiate DNA replication even in cells with incorrect chromosome numbers. Such p53 deficiency and SAC accumulation may cause polyploidization and the emergence of cells with >4N DNA content. Polyploidy is considered protumorigenic because it leads to chromosomal instability that can contribute to tumor development 17–28. Thus, our results may suggest a novel and critical role of p53 in suppressing tumorigenesis-associated DNA aneuploidy under OS.

BubR1 knockdown significantly decreased the emergence of polyploid cells under OS in p53-depleted cells (Fig. 3B and C-d). Insufficient BubR1 is known to lead to the inactivation of SAC function, resulting in an increased incidence of DNA aneuploidy 40–41. Our result is consistent with these reports in that BubR1 downregulation itself increased the numbers of binucleate cells in p53-depleted cells under normal culture conditions (Fig. 3E). However, our results also suggest that BubR1 downregulation suppresses the emergence of polyploid cells caused by OS when p53 is suppressed (Fig. 3C-b). Interestingly, Mad2 downregulation did not suppress the emergence of polyploid cells by OS when p53 was suppressed (Fig. S3). Although the underlying mechanism remains unclear, a new role of BubR1 other than SAC was recently reported by Baker et al. 42, who demonstrated that the reduction in BubR1 levels causes cell senescence by activating the p16Ink4a–Rb signaling pathway in a mouse model. Miyamoto et al. 43 also demonstrated that BubR1 is required for the ubiquitin-mediated proteasomal degradation of CDC20 in the G0 phase and the maintenance of APC/C^CDH1^ activity. Such novel functions of BubR1 may contribute to the phenotypes observed in this study.

In clinical gastric cancer specimens, we found that BubR1 expression level was strongly correlated with p53 dysfunction (Table 1) and the coexistence of p53 dysfunction and high expression of BubR1 was significantly accompanied by DNA aneuploidy (Table 2). These results suggest that the p53 signaling pathway may also regulate BubR1 expression under OS in vivo and the failure of this regulation may contribute to the increase in DNA aneuploidy. An increase in OS was observed in aneuploid cells, and ROS accumulation could play a role in maintaining aneuploidy formation 17–26. The stomach is a digestive organ subjected to OS directly or indirectly through the diet, and some gastric organic disorders, including cancer, could be related to such stress 30. Therefore, the cumulative effects of OS on aneuploid cells could promote gastric carcinogenesis.

**Figure 4:**
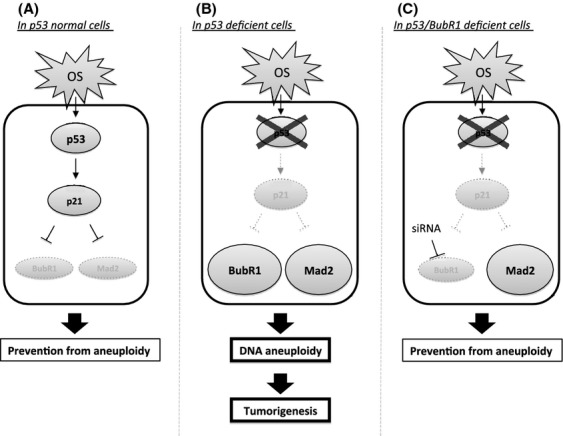
A possible model of gastric carcinogenesis associated with DNA aneuploidy is depicted. (A) When p53 is fully functional, OS does not evoke polyploidization. p53-dependent BubR1 and Mad2 downregulation could be one of the factors contributing to the suppression of polyploidization. (B) In contrast, p53 deficiency may lead to the failure of BubR1 and Mad2 downregulation and OS-induced aneuploidy. (C) BubR1 itself may contribute to the enhancement of OS-induced polyploidization because siRNA-mediated downregulation of BubR1 and not of Mad2 could suppress OS-induced polyploidization. Consistent with this finding, the coexistence of p53 dysfunction and high expression of BubR1 are frequently observed in gastric cancer specimens with aneuploidy, which suggests their involvement in gastric carcinogenesis associated with DNA aneuploidy.

In summary, when p53 is fully functional, OS does not evoke polyploidization. p53-dependent BubR1 and Mad2 downregulation could be one of the reasons for suppressing polyploidization (Fig. 4A). In contrast, p53 deficiency may lead to the failure of BubR1 and Mad2 downregulation, and accumulated BubR1 or Mad2 could contribute to the enhancement of binucleation and polyploidization, which would be the trigger for chromosome missegregation and aneuploidy and, possibly, a promoter of tumorigenesis (Fig. 4B). Importantly, BubR1 downregulation could suppress OS-induced binucleation and polyploidization even in p53-depleted cells (Fig. 4C), suggesting the possibility of BubR1 as a molecular target for the prevention of tumorigenesis through p53 dysfunction and OS-induced aneuploidy. Thus, our findings may have clinical implications for the suppression of gastric carcinogenesis associated with DNA aneuploidy.

## Supporting Information

**Figure S1**. Expression of p53 and BubR1 in gastric cancer. **Figure S2**. Response to KBrO3 in gastric cancer cell lines with or without p53. **Figure S3**. Suppression of Mad2 expression and OS-induced polyploidization in p53-depleted cells.
