# Supplementary material for: Contribution of BubR1 to oxidative stress-induced aneuploidy in p53-deficient cells
Source: Cancer Med. 2013 Jun 26;2(4):447–56. doi: 10.1002/cam4.101 (PMC3799279; doi:10.1002/cam4.101)

Supplementary Fig.1

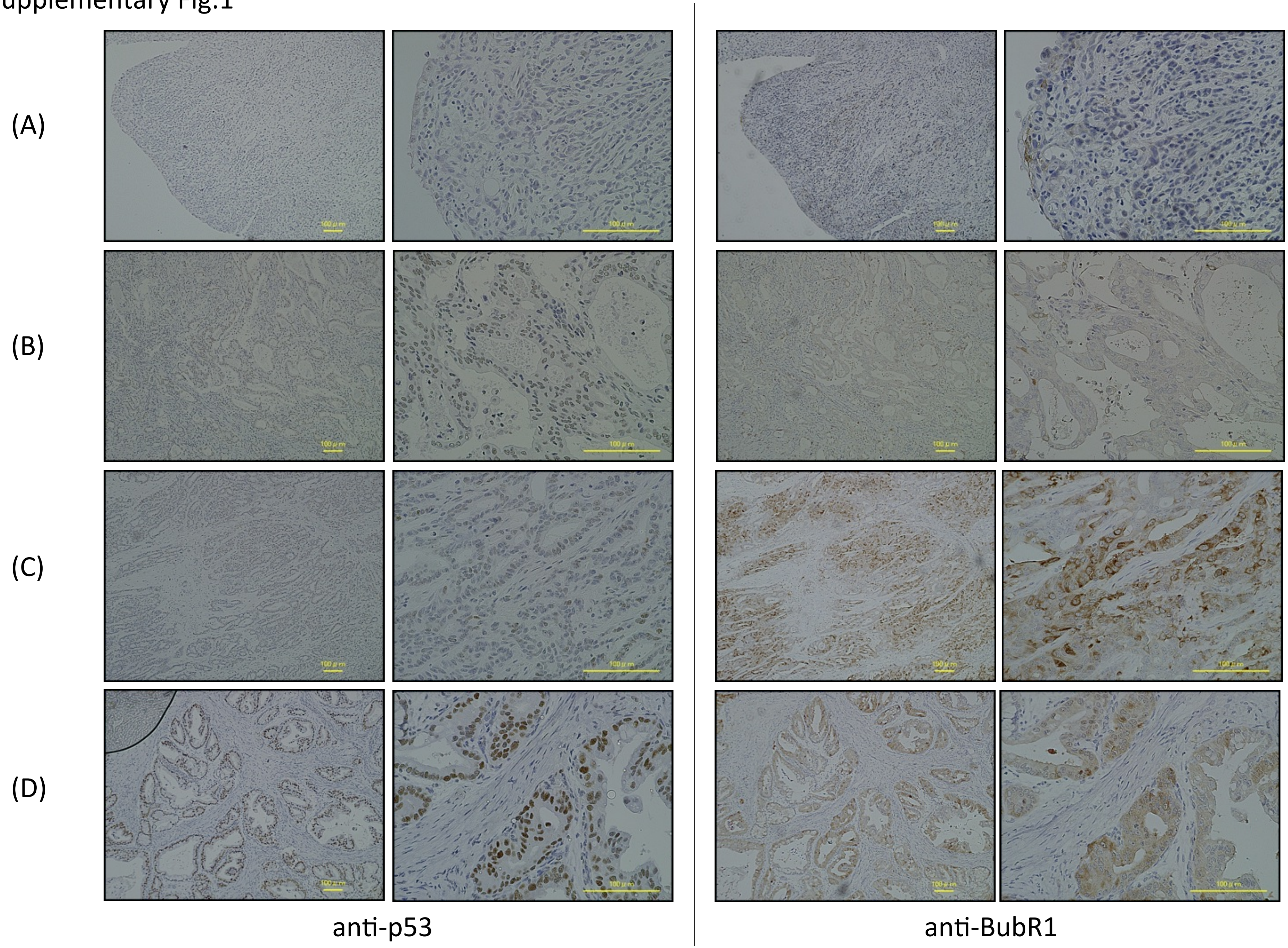

Supplementary Fig.2

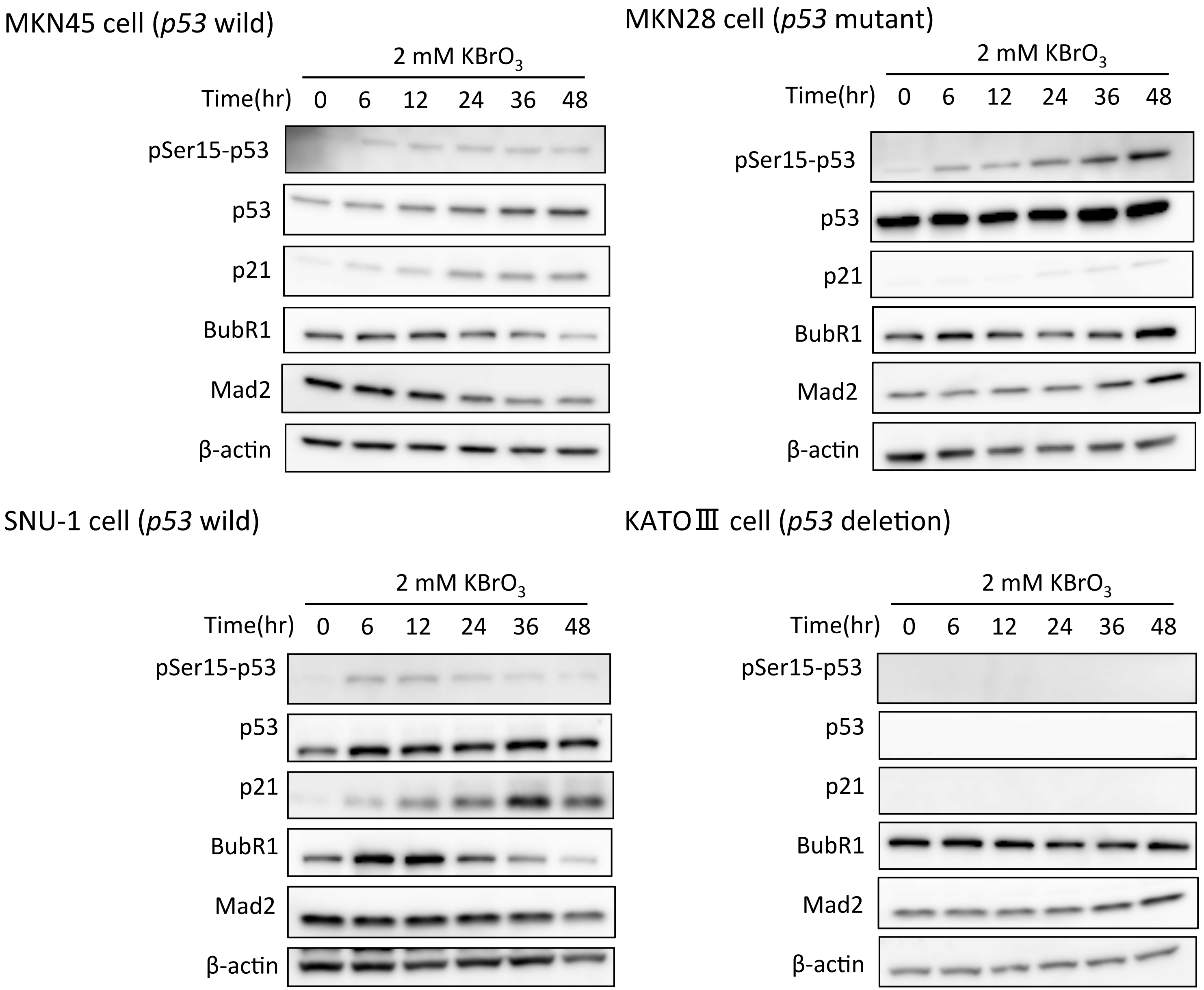

Supplementary Fig.3

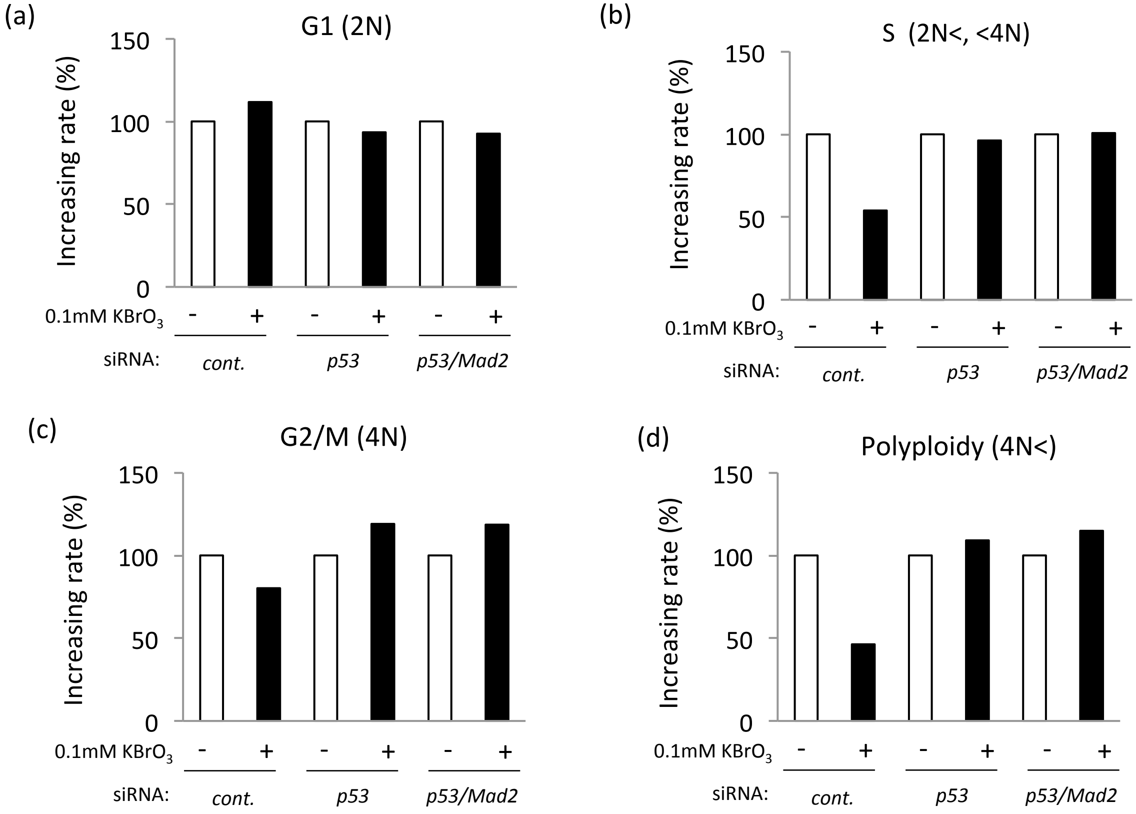

Supplement: Supplementary file 1 — Figure S1. Expression of p53 and BubR1 in gastric cancer. Figure S2. Response to KBrO3 in gastric cancer cell lines with or without p53. Figure S3. Suppression of Mad2 expression and OS-induced polyploidization in p53-depleted cells. [file cam40002-0447-SD1.pdf]
